# Single-cell transcriptomic profiling of halo nevi and normal nevi reveals CD8^+^ T cell activation in melanocytic autoimmunity

**DOI:** 10.3389/fimmu.2026.1771401

**Published:** 2026-05-14

**Authors:** Wenxuan Gao, Haozhen Lv, Yuhua Xie, Ying Chang, Bin Li, Jianmin Chang, Kailv Sun, Ting Chen

**Affiliations:** 1 College of Life Sciences, Beijing Normal University, Beijing, China; 2 National Institute of Biological Sciences, Beijing, China; 3 Department of Dermatology, National Center for Gerontology; National Clinical Research Center for Gerontology; The Key Laboratory of Geriatrics of NHC; Institute of Geriatric Medicine, Chinese Academy of Medical Sciences, Beijing, China; 4 Tsinghua Institute of Multidisciplinary Biomedical Research, Tsinghua University, Beijing, China

**Keywords:** autoimmune disease, halo nevi, normal nevi, single-cell RNA sequencing, CD8+ T cells, melanocytes, antigen presentation, interferon

## Abstract

Halo nevus is an autoimmune skin disorder characterized by autoreactive CD8^+^ T cells that target and destroy melanocytes, resulting in a depigmented perilesional halo. Yet the upstream triggers of this activation remain unclear. Here, we performed single-cell RNA sequencing (scRNA-seq) on halo nevi and normal nevi, identifying ten canonical skin cell types and five melanocyte subclusters. Integrated scRNA-seq and immunofluorescence staining revealed that halo nevi, unlike normal nevi, exhibit robust infiltration of activated, type II interferon-responsive CD8^+^ T cells. Among melanocyte subclusters, one subset (Cluster 3) showed pronounced upregulation of antigen-presenting molecules, interferon-stimulated genes, and chemokines. Comparative analyses further demonstrated that melanocytes in halo nevi increased oxidative phosphorylation, interferon-driven pathways, and antigen processing and presentation. Together, these data provide single-cell transcriptional atlas of halo nevi versus normal nevi, reveal melanocyte-intrinsic and immune-mediated mechanisms underlying autoimmune melanocyte destruction, and highlight pathogenic pathways shared with vitiligo that may inform the development of targeted therapies.

## 1 Introduction

**Figure 1:**
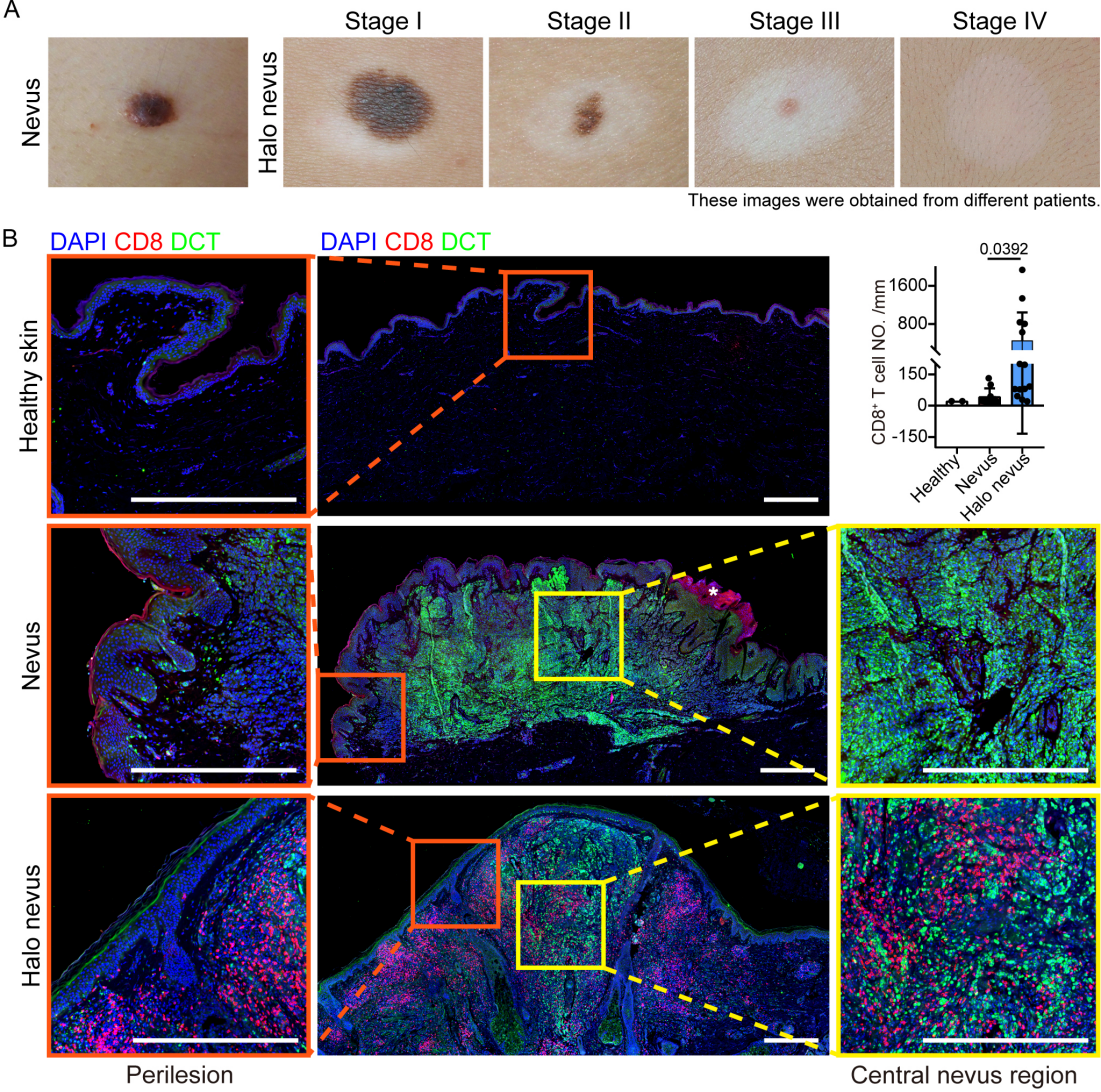
Clinical features and CD8^+^ T cell infiltration in halo nevi and normal nevi. **(A)** Representative clinical images of skin from patients with a nevus and from patients with halo nevi at four different stages. **(B)** Representative immunofluorescence images and quantification of CD8^+^ T cells in healthy skin (n=2) and in the skin of patients with halo nevi (n=14) or normal nevi (n=10). The asterisk (*) indicates areas of non-specific staining. Scale bar: 500 μm. Mean ± SD are presented with p value using unpaired two-tailed Student’s t test.

Halo nevus is a relatively common autoimmune skin disorder with a prevalence of approximately 1%, occurring most frequently in children and adolescents (1, 2). It is clinically characterized by a sharply demarcated, depigmented halo surrounding a central melanocytic nevus (3–7). Mean diameters of central and halo were 5.22 mm (range 3–10) and 12.06 mm (range 5–19), respectively (8, 9). The clinical development of halo nevus is typically divided into four stages. Stage I is defined by the emergence of a depigmented halo around the nevus. Stage II involves progressive fading of pigmentation within the central nevus, often accompanied by erythema. Stage III is marked by partial or complete regression of the nevus itself. Stage IV is characterized by persistent depigmentation that may remain for years before spontaneous repigmentation occurs (**Figure 1A**) (4, 6, 7).

Halo nevi exhibit histopathological and immunological features that closely parallel those observed in vitiligo. In both conditions, melanocyte loss is driven by cytotoxic CD8^+^ T cells that produce effector molecules such as granzyme B (GZMB), perforin, and interferon-γ (IFN-γ) within lesional skin, and the chemokine axis CXCL10–CXCR3 is markedly upregulated (10, 11). Clinical observations further highlight the relationship between the two disorders: 1–48% of individuals with vitiligo also present with halo nevi, and they are frequently considered an early or associated clinical sign of vitiligo (6, 12–16).

Despite these similarities, the upstream mechanisms responsible for aberrant CD8^+^ T-cell activation in both diseases remain poorly defined. Studying halo nevus therefore offers a unique opportunity to identify melanocyte-intrinsic and microenvironmental cues that initiate cytotoxic immune responses in depigmenting autoimmune diseases. In a recent analysis of scRNA-seq datasets from vitiligo and healthy skin, Xu et al. (17) identified a melanocyte subset enriched in vitiligo lesions that exhibited heightened IFN-γ responsiveness and immune activation signatures; the abundance of this subset correlated with disease progression, suggesting a potential role as an upstream modulator of T-cell–mediated melanocyte destruction.

Both halo nevi and normal nevi contain abundant melanocytes within nevus cell nests; however, only halo nevi show prominent T-cell infiltration (18, 19). This divergence provides a powerful comparative framework for dissecting melanocyte-intrinsic properties that may shape local immune activation. Prior studies indicate that melanocytes in nevus nests display features of cellular senescence, and accumulating evidence suggests that senescent cells can promote antitumor immunity (18, 20, 21). These findings raise the possibility that nevus cells have immunoregulatory capacity. Transcriptional or functional differences in melanocytes may contribute to the distinct immune microenvironments.

However, current studies of halo nevi rely primarily on histology and bulk RNA sequencing of whole skin, approaches that obscure cell-type–specific transcriptional heterogeneity. As a result, the cellular diversity and melanocyte subsets that may drive immune activation in halo nevi remain largely uncharacterized.

In this study, we used single-cell transcriptomic profiles from patients with halo nevi or normal nevi to delineate their cellular landscapes. Specifically, our analysis identified ten major cell types and revealed a markedly activated state in CD8^+^ T cells within halo nevi. Furthermore, our investigation of melanocytes uncovered a subset that closely resembles the highly immune-responsive melanocytes previously described in vitiligo. Notably, comparative analysis demonstrated that melanocytes in halo nevi exhibit enhanced antigen presentation, amplified interferon responses, and increased oxidative phosphorylation relative to normal nevi. Collectively, our findings map the distinct transcriptional landscapes of halo nevi and normal nevi, unveiling molecular drivers that may trigger the activation of T cells in skin autoimmune diseases.

## 2 Method

### 2.1 Human skin samples

**Figure 2:**
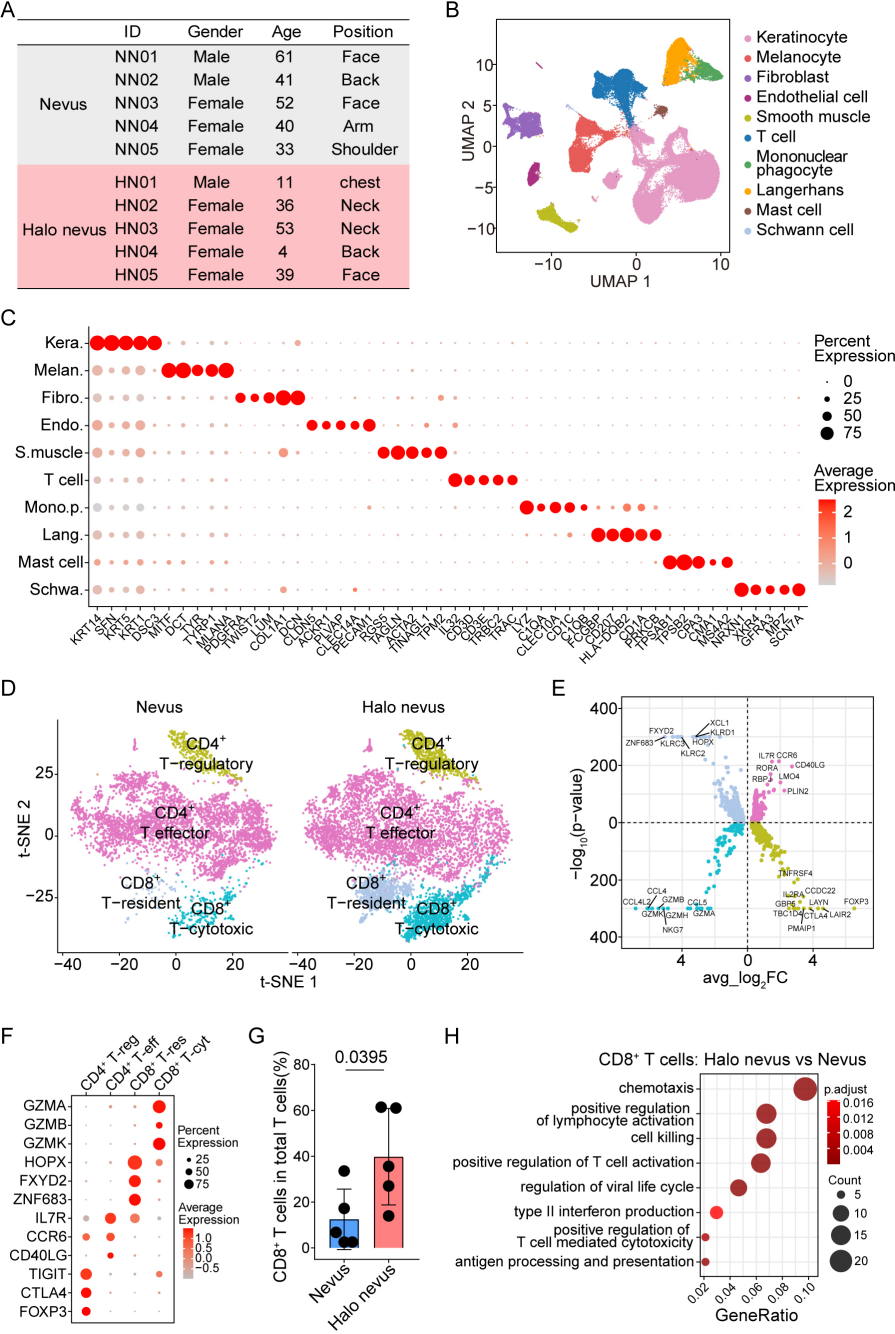
Single-cell landscape of halo nevi and normal nevi highlights an enrichment of activated CD8^+^ T cells. **(A)** Clinical information of donors included in single-cell analysis. **(B)** UAMP visualization of all collected cells showing ten main cell types. **(C)** Dot plot analysis of signature genes for each cell type. **(D)** t-SNE visualization of T cells from patients with halo nevi and normal nevi. **(E)** Volcano plots showing upregulated DEGs in each T cell subtype. **(F)** Dot plot analysis of signature genes for each T cell subtype. **(G)** Percentage of CD8^+^ T cells in total T cells from the skin of patients with halo nevi or normal nevi. Mean ± SD are presented with p value using unpaired two-tailed Student’s t test. n=5 for each group. **(H)** GO analysis of genes enriched in CD8^+^ T cells from halo nevi compared to normal nevi.

This study was approved by the Ethics Committee of Beijing Hospital (2024BJYYEC-KY328-01). For scRNA-seq, we enrolled five halo nevus patients (4 females, 1 male; median age 36, range 4–53) and five nevus patients (3 females, 2 males; median age 41, range 33–61), with clinical details summarized in **Figure 2A**. Additionally, between April 2017 and October 2025, we collected 14 halo nevi, 10 nevi, and 2 healthy control skin samples. These samples were formalin-fixed and paraffin-embedded (FFPE) for immunofluorescent staining.

### 2.2 Single-cell collection from human skin biopsies

Skin biopsies were collected and immediately transferred into sterile, ice-cold phosphate buffer saline (PBS) after surgical operations. Processing of the tissue and subsequent single-cell purification started within 3 hours after biopsy collection. For cell isolation, subcutaneous fat was first carefully removed. The remaining skin tissue was then cut into 1 mm × 1 mm pieces with a surgical scalpel and transferred, with the dermal side down, into 4 mL of 2.4 U/mL dispase (Gibco 17105041) in PBS, followed by incubation at 37 °C with shaking at 80 rpm for 60 minutes.

After incubation, the epidermis was separated from the dermis. The isolated epidermis was digested in 0.25% trypsin–EDTA (Gibco 25200056) at 37 °C for 10 min, neutralized with ice-cold 5% fetal bovine serum (FBS, Gibco 10099141C) in PBS, and dissociated into a single-cell suspension via repeated pipetting and 40-μm filtration. Simultaneously, the dermis was digested in collagenase I (2 mg/mL in HBSS, Sigma C2674) at 37 °C for 1 h with shaking (80 rpm), followed by neutralization and vigorous pipetting through a 40-μm strainer. All cells were centrifuged at 450 g, washed, and resuspended in ice-cold 5% FBS/PBS. Epidermal cells were stained with anti-human CD45 (BD 555485, dilution 1:300) and anti-human CD117 (eBioscience, Cat #12-1178-41, dilution 1:300) for 20 minutes, while dermal cells were stained with anti-human CD45 (dilution 1:300) for 20 minutes. Based on fluorescence-activated cell sorting (FACS) analysis, distinct cellular subtypes were sorted into PBS containing 1% bovine serum albumin (BSA) to minimize cell loss and adhesion, including total immune cells (CD45^+^), epidermal melanocytes (CD117^+^), other epidermal cells (CD45^-^CD117^-^), and dermal non-immune cells (CD45^-^). The sorted populations were then pooled at a ratio of 40:30:30 (immune and epidermal melanocyte: dermal cell: epidermal cell) for downstream single-cell analysis.

### 2.3 Single-cell RNA library construction and sequencing

Single-cell cDNA libraries were prepared and sequenced by GENEWIZ, Inc. Suzhou. In brief, cells were loaded onto a Chromium Single Cell Controller (10x Genomics) using the Chromium Single Cell 3’ Reagent Kit v3 Chemistry, with the HN03 sample processed using the 5’ v2 Chemistry (10x Genomics), according to the manufacturer’s instructions. All libraries were sequenced on the NovaSeq Sequencing System (Illumina). Raw sequencing data were initially processed using Cell Ranger 7.0.0 and aligned to the 10× Genomics human reference genome (refdata-gex-GRCh38-2020-A).

### 2.4 scRNA-seq data analysis and cell-type identification

Seurat V5 was used to further analyze the scRNA-seq data. Cells with fewer than 500 or more than 5,500 detected genes, fewer than 1,000 total UMI counts, or mitochondrial gene percentages exceeding 15% were excluded during quality control. Doublets were removed using the scDblFinder tool. A total of 96,914 cells were used for downstream bioinformatic analyses. Gene expression values were normalized using Seurat’s “NormalizeData” function, and highly variable genes were identified via “FindVariableFeatures”. The normalized data were then scaled with the “ScaleData” function, where mitochondrial gene percentage was regressed out to mitigate technical variation. This was followed by principal component analysis (PCA) implemented with “RunPCA”. Harmony in Seurat was used to integrate data for batch effect removal. These Harmony-corrected embeddings were subsequently used for all downstream analyses: a nearest-neighbor graph was constructed based on the top 40 principal components (PCs), and cellular clustering was performed using the “FindClusters” function. Two-dimensional (2D) embeddings were generated via Uniform Manifold Approximation and Projection (UMAP) and t-distributed Stochastic Neighbor Embedding (t-SNE) to visualize cellular heterogeneity across all samples.

Cluster marker genes were identified using the “FindAllMarkers” function by Wilcoxon rank-sum test with the following parameters: min. pct = 0.25, logfc.threshold = 0.25. Based on previously published marker genes from human skin scRNA-seq studies, cell type annotation was performed, leading to the identification of ten distinct cell types (17, 22, 23). For subtype cell clustering, T cells and melanocytes were extracted and re-clustered separately by Seurat.

### 2.5 Differentially expressed genes and functional enrichment analysis

Differential expression analysis between halo nevi and normal nevi was performed using “FindMarkers” function from Seurat, with the following parameters: min.pct=0, logfc.threshold = 0. Subsequently, all gene symbols were standardized to the latest Human Gene Nomenclature Committee (HGNC) annotations using “checkGeneSymbols” function from the HGNChelper, and corresponding Entrez IDs were retrieved by ID mapping with AnnotationDbi and the human gene annotation database org.Hs.eg.db. Genes with pct.1 > 0.1, |avg_logFC| > 0.25 and p_val_adj < 0.05 were considered as differentially expressed genes. Gene Ontology (GO) analysis of upregulated differentially expressed genes (DEGs) (avg_logFC > 0.25) was performed using clusterProfiler and visualized with the ggplot2.

### 2.6 Cell-cell communication analysis

Cell-cell communication analysis was conducted as previously described using the CellChat R package (v1.6.1) (24). Normalized data from Seurat were input for CellChat analysis. A minimum group size of 10 cells was required to ensure robust interaction inference. Based on the Secreted Signaling category from CellChatDB.human database, CellChat was applied to evaluate intercellular communication networks. Cell-cell interactions were visualized using the “netVisual_circle” function.

### 2.7 Immunofluorescence and image analysis

Formalin-fixed, paraffin-embedded tissue samples from two healthy skin, fourteen patients with halo nevi and ten patients with normal nevi were sectioned at 5 μm thickness. Sections were deparaffinized in xylene, followed by rehydration through a graded ethanol series and rinsed in water for 3 minutes. Endogenous peroxidase activity was quenched with 3% hydrogen peroxide (H_2_O_2_) at room temperature for 10 minutes. Sections were then washed in PBS for 5 minutes and permeabilized in 0.3% H_2_O_2_ in methanol at −20 °C for 30 minutes. Antigen retrieval was performed using Tris-EDTA buffer (pH 9.0) with heat treatment. After three washes in PBS (15 minutes each), sections were blocked for 1 hour at room temperature in blocking buffer containing 2% normal donkey serum, 1% BSA, and 0.3% Triton X-100 in PBS. Sections were subsequently incubated overnight at 4 °C with primary antibodies: anti-human CD8 (Thermo Fisher Scientific, Cat# MA5-16345, dilution 1:500) and anti-human DCT (generated in the T.C. laboratory, dilution 1:3000) diluted in blocking buffer. Following primary incubation, sections were incubated with appropriate fluorescent secondary antibodies at room temperature for 1 hour. Imaging was performed using a Nikon AX confocal microscope with a 20× objective. Z-stacks were acquired at a resolution of 1,024 × 1,024. Images were analyzed using Imaris software (Bitplane), and final figures were assembled and processed in Adobe Illustrator.

## 3 Result

### 3.1 CD8^+^ T cell infiltration and loss of melanocytes characterize halo nevi compared with normal nevi

To investigate the mechanisms underlying the distinctive architecture of halo nevi—characterized by a pigmented central nevus surrounded by a depigmented halo—we first examined skin biopsies from healthy individuals (n=2) and from patients with either halo nevi (n=14) or normal nevi (n=10). Immunofluorescence staining using DCT as a melanocyte-specific marker confirmed abundant melanocytes within both halo nevi and normal nevi. Similarly, these melanocytes were predominantly organized into well-defined nevus nests. Compared to healthy skin and normal nevi, melanocytes were largely absent from the epidermis encircling the central nevus in halo nevi, consistent with the clinical manifestation of perifocal depigmentation (**Figure 1B**). Staining of CD8^+^ T cells revealed that halo nevi displayed pronounced infiltration of CD8^+^ T cells, whereas healthy skin and normal nevi contained few even no CD8^+^ T cells. Notably, infiltrated CD8^+^ T cells in halo nevi samples were not restricted to the perilesional region but also penetrated deeply into the nevus cell nests. Together, these observations indicate that halo nevi displayed a heightened local immune response relative to normal nevi and suggest that nevus melanocyte-CD8^+^ T cell interaction may contribute to the disease progression.

### 3.2 Single-cell analysis revealed the transcriptional landscape in halo nevi and normal nevi

To characterize cell-type–specific transcriptional differences between halo nevi and normal nevi, we performed single-cell RNA sequencing (scRNA-seq) on skin biopsies obtained from five patients with halo nevi and five with normal nevi (**Figure 2A**; **Supplementary Figures 1A, B**). Following stringent quality-control filtering to remove low-quality cells and doublets, a total of 96,914 high-quality cells were retained for downstream analysis. Unsupervised clustering and annotation based on canonical marker genes identified ten major cell types: keratinocytes, melanocytes, fibroblasts, endothelial cells, smooth muscle cells, T cells, mononuclear phagocytes, Langerhans cells, mast cells, and Schwann cells (**Figure 2B**; **Supplementary Figure 1C**; **Supplementary Table 1**). For each cell type, representative markers were confirmed and visualized using dot plots, heatmap, violin plots, and feature plots to illustrate their distinct transcriptional signatures (**Figure 2C**; **Supplementary Figure 2**). Overall, both halo nevi and normal nevi harbored the same major cellular constituents, providing a robust foundation for subsequent comparative analyses of cell-type–specific transcriptional states.

### 3.3 scRNA-seq analysis identifies activation of CD8^+^ T cells in halo nevi

To further delineate immunological differences between halo nevi and normal nevi, we analyzed 12,880 T cells captured in our dataset. Unsupervised clustering identified four major T-cell subsets based on canonical transcriptional signatures: CD8^+^ cytotoxic T cells, CD8^+^ tissue-resident memory T cells, CD4^+^ regulatory T cells, and CD4^+^ effector T cells (**Figure 2D**; **Supplementary Table 2**). Marker genes characteristic of each subset were visualized using volcano plots and dot plots, confirming the robustness of subtype classification (**Figures 2E, F**). Quantitative comparison of T-cell composition revealed a striking increase in the proportion of CD8^+^ T cells within the total T-cell compartment in halo nevi relative to normal nevi (**Figure 2G**).

We next performed differential gene expression analysis on CD8^+^ T cells to investigate their activation state. A total of 293 differentially expressed genes (DEGs) were identified between halo nevi and normal nevi, with 259 genes significantly upregulated in halo nevi (**Supplementary Table 3**). GO enrichment analysis demonstrated that upregulated genes were strongly associated with pathways involved in T-cell activation, antigen processing and presentation, cytolytic function, and type II interferon–mediated responses (**Figure 2H**). These findings collectively indicate that CD8^+^ T cells in halo nevi exhibit a markedly heightened activation profile compared with those in normal nevi, highlighting their role in melanocyte destruction and halo formation.

### 3.4 scRNA-seq uncovers the heterogeneity of melanocyte subclusters

**Figure 3:**
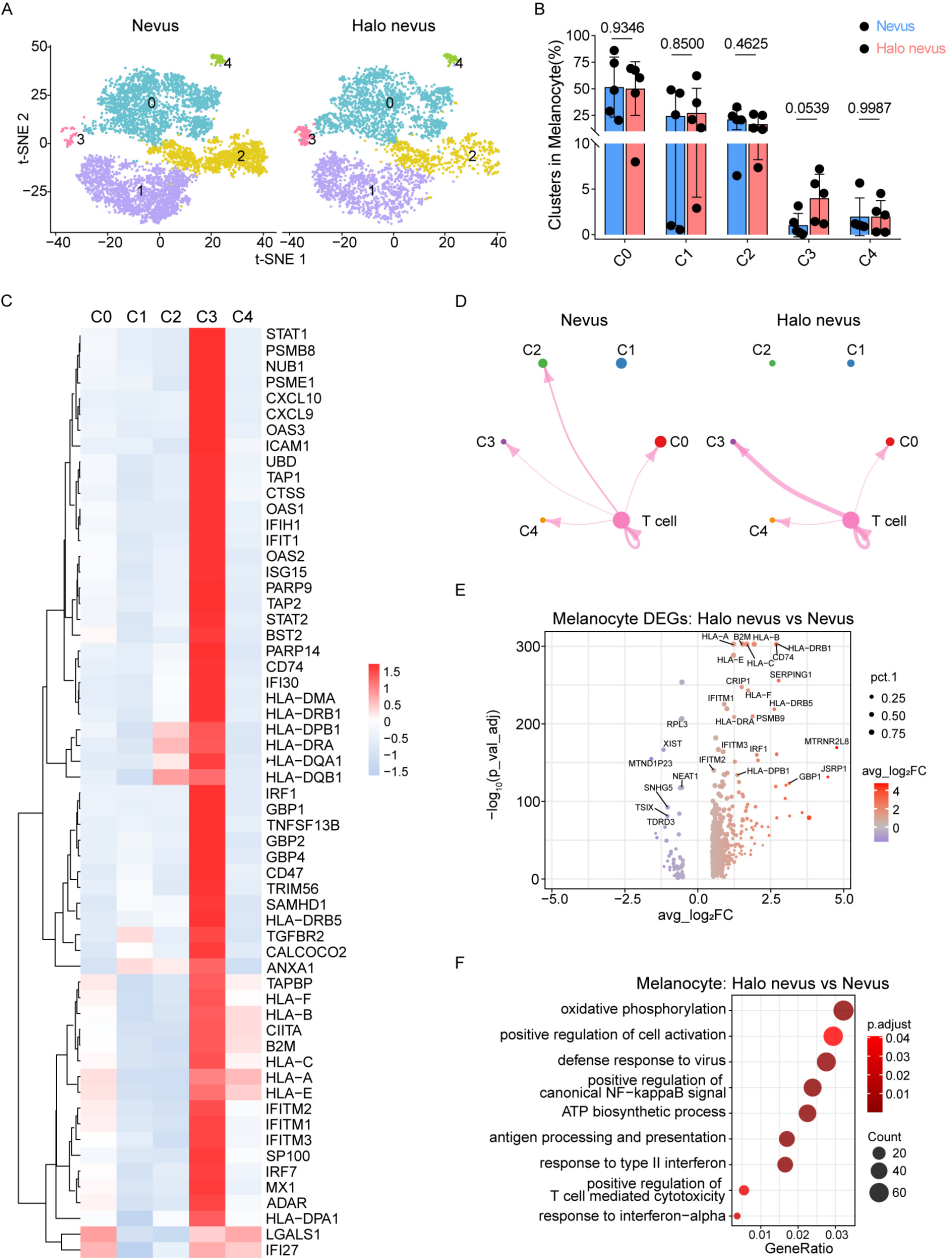
Melanocytes in halo nevi exhibit an immune activation signature. **(A)** t-SNE visualization showing the distribution of five melanocyte subclusters from halo nevi and normal nevi samples. **(B)** Analysis of the composition of melanocyte subclusters. Mean ± SD are presented with p value using unpaired two-tailed Student’s t test. n=5 for each group. **(C)** Heatmap analysis of antigen presentation, interferon response, and T cell activation–associated gene expression in melanocyte clusters. **(D)** Cell–cell communication networks from T cells to melanocyte clusters in halo nevus and nevus skin. **(E)** Volcano plot of DEGs in melanocytes from halo nevi compared to normal nevi. **(F)** GO analysis of genes enriched in melanocytes from halo nevi compared to normal nevi.

Given that loss of melanocytes is a defining histopathological feature of halo nevi, we next investigated transcriptional alterations in melanocytes from halo nevi versus normal nevi. Melanocytes were extracted from the integrated scRNA-seq dataset and resolved into five transcriptionally distinct subclusters (**Figure 3A**; **Supplementary Table 4**). The relative abundance of the remaining subclusters (C0, C1, C2, and C4) was comparable between groups and the proportion of C3 melanocytes show an increasing trend in halo nevi compared with normal nevi (p = 0.0539) (**Figure 3B**). GO analysis of cluster-specific marker genes revealed that C3 was uniquely enriched for pathways related to interferon responses and antigen processing and presentation (**Supplementary Figure 3**).

Consistent with the pathway-level enrichment, heatmap analyses demonstrated that C3 melanocytes exhibited robust upregulation of genes involved in antigen presentation—including multiple HLA class I and II components, B2M, and CD74—as well as interferon-stimulated genes such as STAT1, IRF1, IRF7, IFIH1, OAS1, and ISG15. Importantly, the chemokines CXCL9 and CXCL10, which are key mediators of CD8^+^ T cell recruitment and have established roles in vitiligo pathogenesis, were also prominently elevated in the C3 subcluster (**Figure 3C**).

To investigate potential crosstalk between melanocytes and T cells, we applied the CellChat algorithm to infer ligand–receptor communication networks. Interactions from T cells to C3 melanocytes were markedly stronger in halo nevi than in normal nevi, suggesting that C3 melanocytes may be preferentially targeted by, or engaged with, infiltrating T cells in halo nevi (**Figure 3D**).

We further performed direct differential expression analysis comparing all melanocytes from halo nevi and normal nevi, identifying approximately 2,430 genes upregulated in halo nevi (**Figure 3E**; **Supplementary Table 5**). In addition, DEGs analysis were conducted for all other major cell types, while Schwann cells were excluded from this analysis due to their low cell numbers (**Supplementary Table 6**). GO enrichment analysis of the genes upregulated in halo nevi compared to normal nevi melanocytes revealed pronounced activation of pathways involved in oxidative phosphorylation, indicating altered metabolic activity; antigen processing and presentation, consistent with enhanced immunogenic signaling; interferon-mediated responses, reflecting activation of innate and adaptive immune programs; and pathways related to T-cell cytotoxicity, further highlighting potential bidirectional interactions between melanocytes and infiltrating CD8^+^ T cells (**Figure 3F**).

Collectively, these results demonstrate that melanocytes in halo nevi adopt a highly immunogenic, metabolically active, and interferon-responsive state. This transcriptional reprogramming is exemplified by the expansion and activation of the C3 melanocyte subcluster and may represent a key upstream driver of local CD8^+^ T cell activation and melanocyte destruction in halo nevi.

## 4 Discussion

In this study, we used single-cell transcriptomic profiling of halo nevi and normal nevi to define their cellular composition and to pinpoint disease-associated transcriptional programs. We identified ten major cell types and found that CD8^+^ T cells in halo nevi adopt a distinctly activated, cytotoxic, and interferon-responsive state that is largely absent in normal nevi. Melanocyte analysis revealed a transcriptionally distinct subset in halo nevi resembling the highly immune-responsive melanocytes described in vitiligo. Comparative analyses showed that melanocytes in halo nevi upregulate antigen-processing and antigen-presentation pathways, exhibit heightened interferon-stimulated gene expression, and increase oxidative phosphorylation relative to melanocytes in normal nevi. These findings refine the cellular architecture of halo nevi and identify melanocyte-intrinsic programs that may sensitize these cells to immune recognition and attack.

Vitiligo and halo nevi are both skin autoimmune diseases that share the clinical hallmark of depigmentation. The loss of melanocytes in halo nevi is generally considered analogous to vitiligo, where CD8^+^ T-cell attack plays a central role. Halo nevi are characterized by dense infiltration of T cells, predominantly CD8^+^ T cells that mirror the vitiligo phenotype. These cells exhibit high expression of granzyme B, IFN-γ, and the activation marker CD69, which collectively drive melanocyte loss (10, 11, 25, 26). The recruitment of T cells to the skin can be mediated by the CXCL10-CXCR3 axes (27–30). In patients with halo nevus or vitiligo, the high cutaneous expression of CXCL10 appears to drive the localization of CXCR3^+^CD8^+^ T cells into the skin, reflected by their decreased frequency in peripheral blood mononuclear cells (11). Despite these insights, the primary upstream triggers that initiate self-antigen recognition and T-cell activation in both conditions remain unknown.

Our study found that while both halo nevi and normal nevi contain abundant melanocytes forming nevus cell nests, normal nevi maintain an immunologically quiescent microenvironment with minimal T-cell infiltration. This stark divergence highlights a critical transition from immune tolerance in normal nevi to immune recognition in halo nevi, implying the presence of melanocyte-intrinsic or microenvironmental cues that unveil or alter antigenic signals to the immune system.

Melanocytic nevi frequently harbor oncogenic mutations such as BRAFV600E, NRASQ61R/K, or HRASG12V, which induce a stable oncogene-induced senescence program rather than malignant transformation (31–35). Although senescent, melanocytes in nevi retain diverse biological activities, including secretion of growth-promoting factors that regulate hair follicle stem cell proliferation (36). Spatial transcriptomic studies demonstrate that melanocytes in halo nevi can enhance dendritic-cell activation and antigen presentation (37). Our work extends these findings by identifying melanocyte subpopulations in halo nevi that display interferon-driven and antigen-presenting phenotypes, suggesting that melanocytes themselves actively shape—and possibly initiate—the inflammatory niche.

Consistent with this model, melanocytes in halo nevi showed elevated expression of key antigen-presentation components, including MHC-I, MHC-II, and B2M. Antigen presentation is central to T-cell recognition (38), and dysregulation of MHC genes is implicated broadly in autoimmune and inflammatory diseases (39). Analogous to melanoma “engagement zones” enriched for B2M that promote CD8^+^ T-cell accumulation and effector activity (40), enhanced antigen presentation in halo nevi likely facilitates T-cell recruitment and cytotoxicity. Similarly, MHC-II upregulation can amplify local CD4^+^ T-cell responses (41). Notably, since IFN-γ is actively secreted during T-cell-mediated destruction of melanocytes, the upregulation of MHC-related molecules can be viewed as a reactive consequence of the immune attack, as these genes are canonical downstream targets of IFN-γ signaling (42). All these changes likely reflect melanocyte exposure to an interferon-rich microenvironment, as type I and type II interferons are potent inducers of antigen-presentation machinery (43).

Together, these findings support a model in which aberrant interferon signaling, enhanced melanocyte antigen presentation, and potentially altered melanocytic antigens collaborate to drive recruitment and activation of cytotoxic T cells, ultimately resulting in melanocyte destruction and the development of the characteristic depigmented halo. Halo nevi therefore provide a valuable natural model for understanding how self-tolerance toward melanocytes is broken—a central question shared across autoimmune and antitumor immunity. Specifically, deciphering the mechanism of halo nevi may reveal triggers for depigmentation in vitiligo, while providing a blueprint for inducing potent immune responses against melanoma.

This study has limitations. The absence of an animal model restricts mechanistic interrogation, and the relatively small patient cohort introduces variability. Because halo nevi likely exist along a dynamic immunological trajectory, our cross-sectional sampling captures only a snapshot of disease evolution. Larger cohorts, longitudinal sampling, and functional perturbation studies will be necessary to define causal pathways.

In summary, this study presents the first single-cell transcriptional landscape of halo nevi and normal nevi. Our findings demonstrate that halo nevi are characterized by the activation of CD8^+^ T cells, driven by melanocytes undergoing enhanced oxidative phosphorylation and displaying high immunogenicity, which facilitates T-cell recognition and attack. These results not only deepen our understanding of halo nevus pathogenesis but also offer novel mechanistic insights into vitiligo and melanoma.

## Data Availability

The data can be accessed through the National Genomics Data Center (NGDC) (https://ngdc.cncb.ac.cn/) under the BioProject accession number: PRJCA054006.

## Supplementary material

Supplementary Figure 1 Experimental workflow for single-cell isolation and UMAP visualization of halo nevi and normal nevi samples. (A, B) Experimental workflow (A) and representative FACS profiles (B) to obtain single cells from human skin biopsies for scRNA-seq. (C) UMAP visualization showing the distribution of cells from halo nevi and normal nevi samples.

Supplementary Figure 2 Validation of major cell-type identification in scRNA-seq data. Heat map (A), violin plot (B) and feature plot (C) analysis of differentially expressed genes in each cell type.

Supplementary Figure 3 The enriched GO terms results of marker genes of five melanocyte subclusters.
