# Supplementary figures and images for: Single-cell transcriptomic profiling of halo nevi and normal nevi reveals CD8+ T cell activation in melanocytic autoimmunity
Source: Front Immunol. 2026 May 14;17:1771401. doi: 10.3389/fimmu.2026.1771401 (PMC13216749; doi:10.3389/fimmu.2026.1771401)

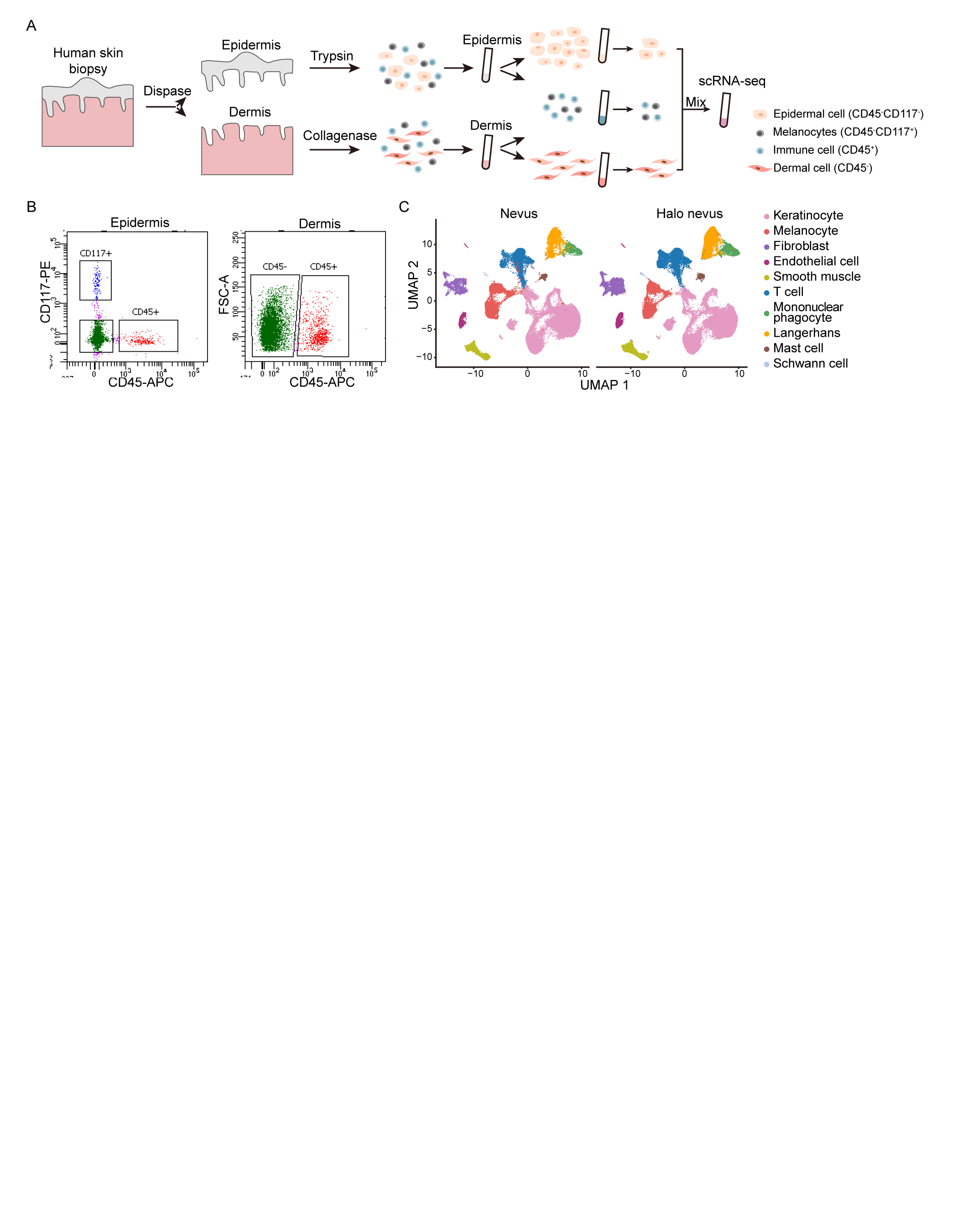

Supplement: Supplementary Figure 1 — Experimental workflow for single-cell isolation and UMAP visualization of halo nevi and normal nevi samples. (A, B) Experimental workflow (A) and representative FACS profiles (B) to obtain single cells from human skin biopsies for scRNA-seq. (C) UMAP visualization showing the distribution of cells from halo nevi and normal nevi samples. [file Image1.jpeg]

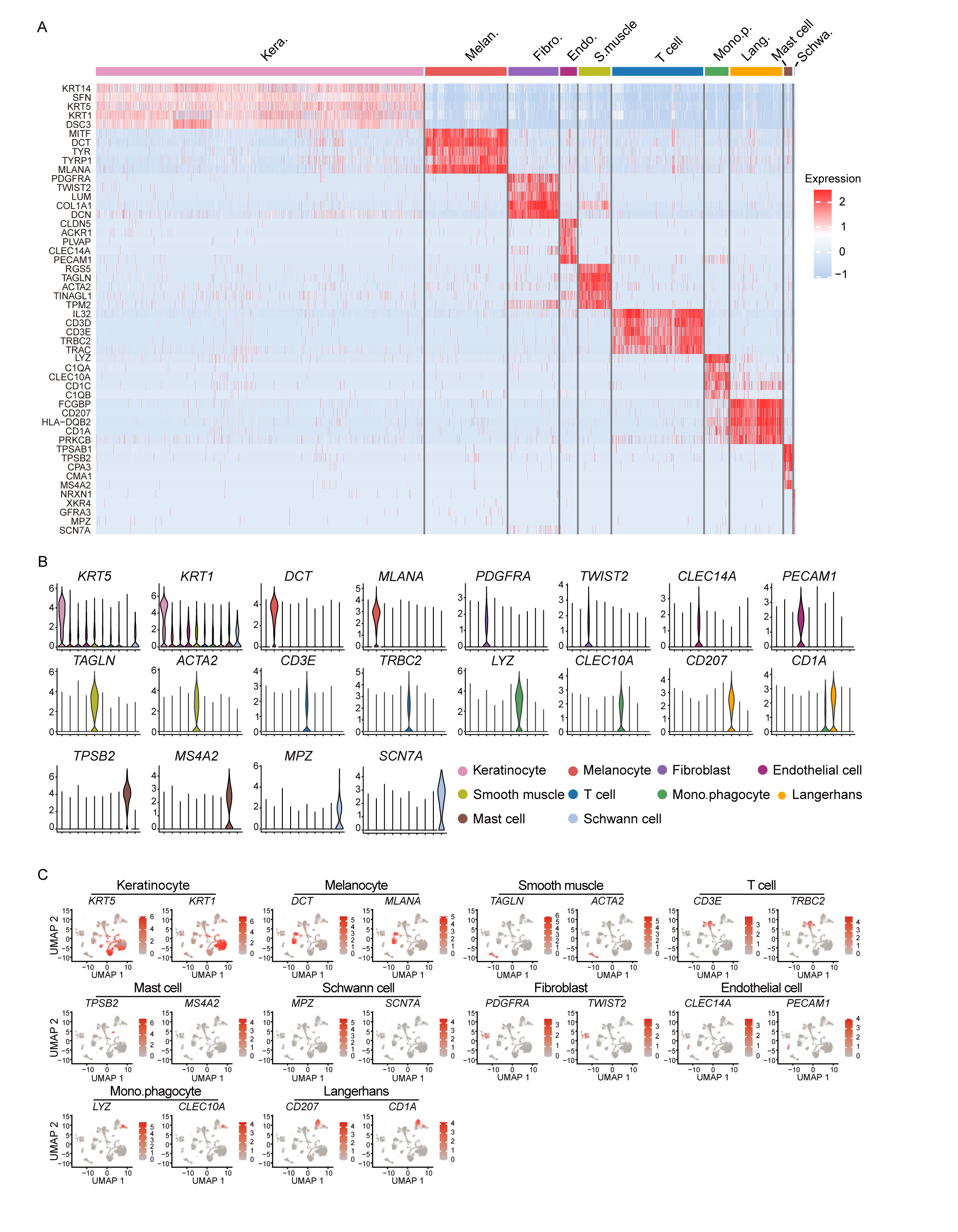

Supplement: Supplementary Figure 2 — Validation of major cell-type identification in scRNA-seq data. Heat map (A), violin plot (B) and feature plot (C) analysis of differentially expressed genes in each cell type. [file Image2.jpeg]

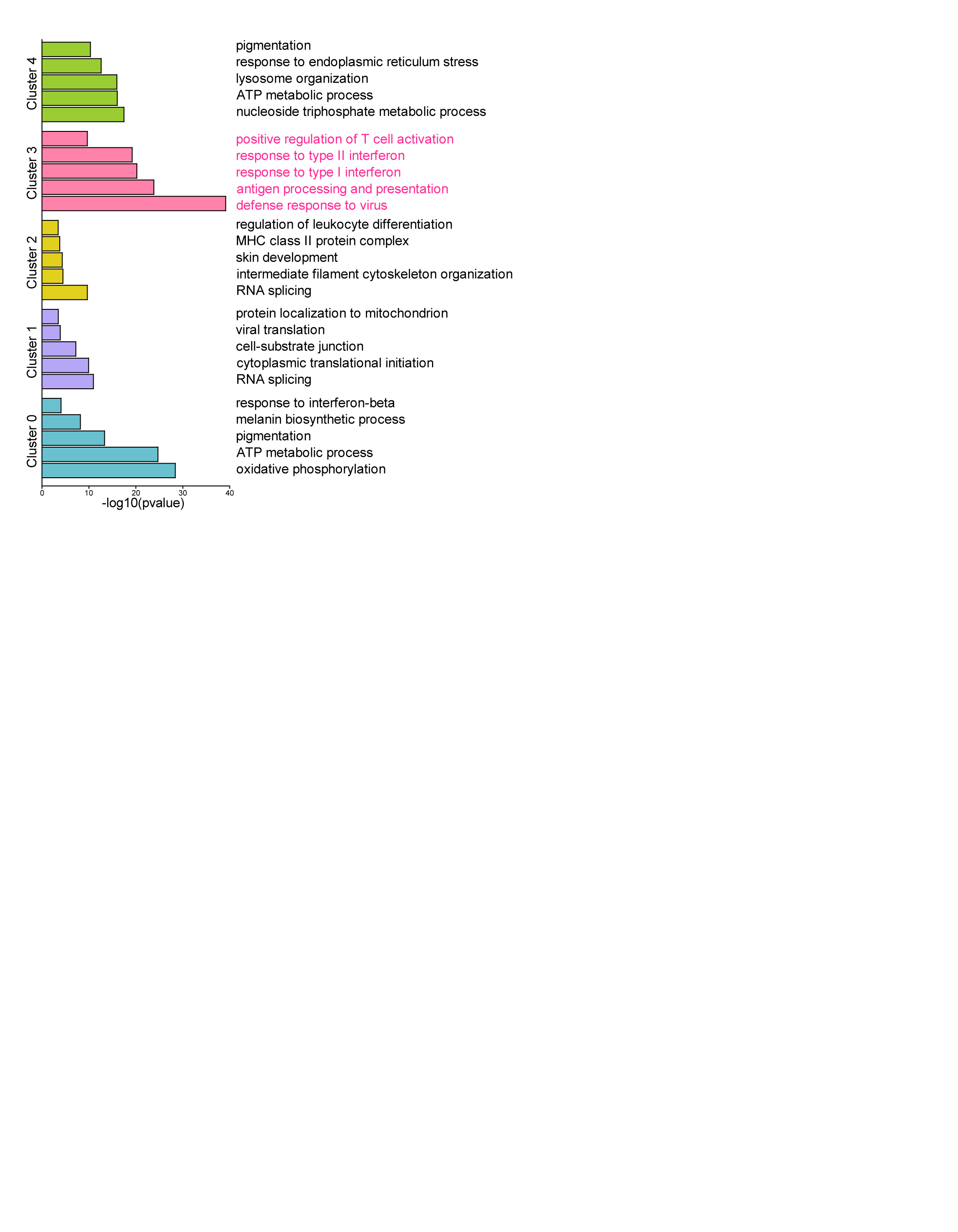

Supplement: Supplementary Figure 3 — The enriched GO terms results of marker genes of five melanocyte subclusters. [file Image3.jpeg]
